# Changing trend analysis on early detection of rifampicin resistant tuberculosis patients in southwestern area of China, 2016–2020

**DOI:** 10.1371/journal.pone.0280578

**Published:** 2023-11-16

**Authors:** Yunbin Yang, Liangli Liu, Xing Yang, Ling Li, Kunyun Lu, Jinou Chen, Zhixiang Xu, Lin Xu

**Affiliations:** 1 Division of Tuberculosis Control and Prevention, Yunnan Center for Disease Control and Prevention, Kunming, China; 2 Family Health International 360 China Kunming Office, Kunming, Yunnan, China; The University of Georgia, UNITED STATES

## Abstract

**Background:**

There were no data about prevention and control status of RR-TB in a poor area with high burden of TB in China. In order to develop evidence-based RR-TB response strategies and improve enrollment of RR-TB patients in Yunnan province, China, this study was aimed at analyzing the changing trends in the detection and enrollment of RR-TB patients and examining the factors that may have implication on enrollment in treatment.

**Methods:**

Data, which includes demographics, screening and testing, and treatment enrollment, was collected from the TB Management Information System. Retrospective data analysis and factors analysis were applied. Descriptive statistics, Chi-square test, Rank sum test and logistic regression analysis were used.

**Results:**

From 2016 and 2018, the province had been challenged by low levels of screening, detection and enrollment of RR-TB. During the period between 2019 and 2020, a comprehensive model of RR-TB prevention and control was established in Yunnan, characterized by a robust patient-centered approach for RR-TB care and multiple, targeted interventions through the cascade of care from detection to treatment. In 2020, 93.8% of the bacteriologically positive TB patients were screened for RR-TB, which had been significantly increased by 146.9% from 38.0% in 2016. The interval from initial consultation at RR-TB facility to diagnosis (inter-quartile range) was reduced from 29.5 (1–118) days in 2016 to 0 (0–7) days in 2020. Despite the increasing rates of enrollment of RR-TB patients over the years, non-enrollment of those detected was still high (32.3%) in 2020. The main reasons for non-enrollment identified were refusal of treatment due to financial difficulties, loss to follow-up or death before starting treatment. Multivariate analysis showed that the elderly patients aged 65 or above (OR = 2.7, CI: 1.997–3.614), new patients (OR = 0.7, CI: 0.607–0.867), conventional DST used for confirmatory diagnosis of RR-TB (OR = 1.9, CI: 1.620–2.344) and diagnosis of RR-TB being conducted by the RR-TB care facilities at the prefecture and municipal level (OR = 4.4, CI: 3.608–5.250) have implications on RR-TB non-enrollment.

**Conclusions:**

As a comprehensive RR-TB model was implemented in Yunnan with scaled up use of molecular test for rapid detection of RR-TB, initial screening of RR-TB were decentralized to the county- and district-level to strengthen rapid, early detection of RR-TB, achieving a higher coverage of screening in the end. However, there remains a major gap in enrollment of RR-TB. The main barriers include: limited knowledge and awareness of RR-TB and financial burdens among patients, delayed diagnosis, loss to follow-up, difficulties in self care and travel for elderly patients, and limited capacity of clinical management at the lower-level RR-TB care facilities. The situation of the RR-TB epidemic in Yunnan could be improved and contained as soon as possible by continuous strengthening of the comprehensive, patient-centered model with targeted interventions coordinated through multi-sectoral engagement to improve enrollment of RR-TB patients.

## Introduction

The World Health Organization (WHO) pointed out in the Global Tuberculosis Report 2020 that there were about 65,000 cases of rifampicin resistant tuberculosis (RR-TB) in China in 2019, accounting for 14% of the world’s total, ranking second in the world. In addition, the RR-TB rates of new and re-treated TB patients in China (7.1% and 23.0%) were higher than the global average (3.3% and 18.0%) [1].

Nearly 74% of Yunnan’s total population (46 million people) live in remote, mountainous rural areas with inconvenient access to transportation. Although Han Chinese people account for 67% of its total population, Yunnan is regarded as one of the most ethnically diverse provinces in China, with a large number of ethnic groups and ethnic minority population. The province covers an area of about 394,000 square kilometers, more than 94% of which is mountainous [2].

Yunnan is one of the provinces with high burden of TB in China. In response to the WHO guideline for TB drug resistance surveillance [3], a baseline survey of TB drug resistance was carried out in Yunnan in 2010. According to the survey, 4.4% of new patients and 22.2% of re-treated patients were estimated to be RR-TB. It was predicted that Yunnan sees approximately 1,000 new cases of RR-TB each year [4]. To find, treat and cure all TB cases, including RR-TB, is a key action in the WHO Stop TB Strategy. However, the data in Yunnan showed that less than 60% of the RR-TB patients were enrolled in RR-TB treatment, which is far below the average levels of the whole country and economically developed coastal provinces in the same period [5–7]. The enrollment of RR-TB is a critical issue to tackle in Yunnan.

This study examined the changes in the detection and enrollment of RR-TB in Yunnan from 2016 to 2020, and analyzed the factors that may have implication on the enrollment of RR-TB, which will provide targeted evidence to inform future development of sound RR-TB response.

## Methods

### Study setting

Yunnan, which lies on China’s southwestern border, is an economically underdeveloped province with 16 prefectures and 129 counties [8], featured by poor health care resources, large populations of native ethnic minorities, and limited public awareness for communicable diseases. Yunnan’s response to RR-TB started late and were, therefore, constrained by a weak foundation at the very beginning. Since the implementation of *the 13*^*th*^ *Five-Year Plan for TB Prevention and Control* in Yunnan in 2016, the provincial government had focused programs and resources to strengthen the RR-TB response. A comprehensive prevention and control model for RR-TB was established to improve the diagnosis, enrollment, treatment and management of RR-TB. One of the major achievements was a patient-centered approach for RR-TB care developed through the Control and Prevention of TB (CAP-TB) project in partnership with FHI 360, which included multiple players. Within the model, the system of Centers of Disease Prevention and Control (CDCs) plays a pivotal role in programmatic management, drug-susceptible TB care facilities are engaged in screening of RR-TB, RR-TB care facilities provide clinical care for patients, and the community-level health care institutions provide continuity of care and support to the patients initiated on treatment. This model entails targeted, evidence-based interventions along the care cascade of RR-TB patients from diagnosis, treatment initiation and follow-up care to ensure treatment success, including expanded use of molecular diagnostic tests for rapid diagnosis of drug resistance, introduction of standardized shorter-course treatment regimen, patient-centered education, counselling and care, use of a smart, electronic system for efficient clinical and case management, and strengthened medical security policies for reduction of financial burdens on RR-TB patients.

### Data collection

Data for this study were retrieved from the Tuberculosis Management Information System (TBMIS), which was launched in Yunnan in January 2005 with the whole province covered. All TB prevention and control institutions in the province initiate real-time data entry to TBMIS for the required TB care information. Through the system, the health professionals from CDCs and RR-TB care facilities could provide real-time registration and update of clinical data of TB patients according to standard operating guidelines [9]. TBMIS collects standardized reports of all cases diagnosed with TB. The TBMIS data used for analysis included demographic information of RR-TB patients (gender, age, ethnicity, occupation, place of residence, type of patient, laboratory tests, diagnostic delay), the date of RR-TB diagnosis, the date of treatment initiation for RR-TB and reasons for non-enrollment.

### Data analysis

SPSS Statistics Version 22.0 was used for analysis of patient data in this study. Descriptive statistics used for data analysis included continuous variables with mean and standard deviation used for normal distribution and median and inter-quartile range for non-normal distribution; categorical variables expressed with absolute and relative frequencies (percentage). The chi-square test for trend was used to analyze the changes for the screening, detection and enrollment of RR-TB over the years from 2016 to 2020. Multivariate rank sum test was run to examine the diagnostic delay of RR-TB. Non-enrollment among RR-TB patients was set as the dependent variable, and logistic regression analysis was performed to identify risk factors for non-enrollment. Variables with *P*-values≤0.2 in the univariate analysis were included in the multivariate analysis, and the models were refined by backward elimination, guided by the changes in log-likelihood for a continuous model at a significance level of 0.05.

### Ethical approval

This retrospective study was consulted to the ethics committees of Yunnan Center for Disease Control and Prevention. We did not include any data of patients’ personal information, including name, identity information, address, telephone number, etc. Therefore ethical consent was not essential.

## Results

### Change trend analysis of RR-TB screening in Yunnan, 2016–2020

In order to rapidly increase screening and detection of RR-TB in the province, from 2016 to 2020, the Yunnan provincial CDC led the provincial efforts of strengthening the testing capacities of TB laboratories of all levels, streamlining the procedure of RR-TB screening, introducing and scaling up use of molecular diagnostic tests for rapid detection of drug resistance, and screening bacteriologically positive TB patients for RR-TB. In 2020, 93.8% of the pulmonary TB patients were screened for RR-TB in Yunnan, which was 190.7% higher than 32.3% in 2017 (χ^2^_trend_ = 65.9, *P*<0.001). As the screening rate increased, the number of RR-TB cases identified also increased year by year, from 309 (30.9%) in 2017 to 703 (70.3%) in 2020 (χ^2^_trend_ = 29.2, *P*<0.001) (Table 1).

**Table 1:** Trend distribution of RR-TB screening and detection in Yunnan, 2016–2020.

Years	Number of bacteriologically positive patients	Number of BP-TB patients screened for RR-TB	RR-TB screening rate (%)*	Number of RR-TB patients detected*
**2016**	6986	2655	38.0	309
**2017**	8311	2682	32.3	343
**2018**	10249	5079	49.6	380
**2019**	14586	11964	82.0	705
**2020**	15324	14375	93.8	703

Bacteriologically positive patients refer to the TB patients who were tested using sputum smear, sputum culture, molecular tests or other laboratory diagnostics with Mycobacterium tuberculosis or its DNA detected.

RR-TB screening: Screening of TB patients for RR-TB by molecular or conventional drug susceptibility testing (DST).

* The trend chi square test showed that RR-TB screening rate and number of RR-TB patients detected in 2016–2020 were statistically different.

**Fig 1:**
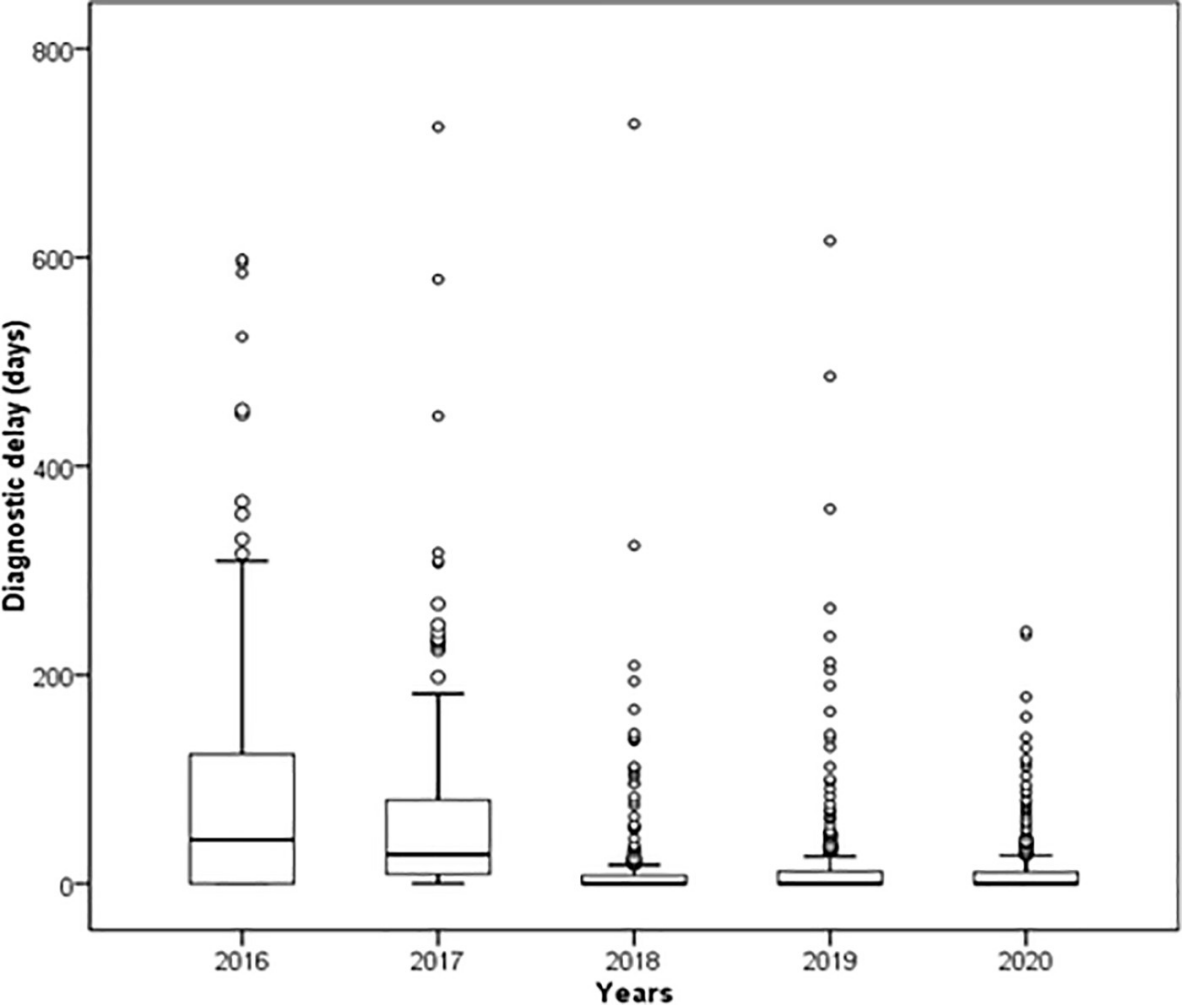
Distribution of diagnostic delay variations for RR-TB in Yunnan, 2016–2020. Diagnostic delay (days) refers to the time interval (number of days) between the first consultation at the RR-TB care facilities to the diagnosis of RR-TB. The figure above showed the median and inter-quartile distribution of days of delay to the diagnosis of RR-TB. * The trend chi square test showed that diagnostic delay in 2016–2020 was statistically different.

Increasing introduction and use of molecular diagnostics for rapid detection of drug resistance at the county level resulted in improved delay to diagnosis of RR-TB in Yunnan year by year, measured as the interval from initial consultation at RR-TB care facilities to diagnosis [10]. For example, the median diagnostic delay (inter-quartile range) was reduced from 29.5 (1–118) days in 2016 to 0 (0–7) days in 2020 (χ^2^ = 265.1, *P*<0.001) (Fig 1).

### Characteristics of enrollment in treatment of RR-TB in Yunnan, 2016–2020

**Fig 2:**
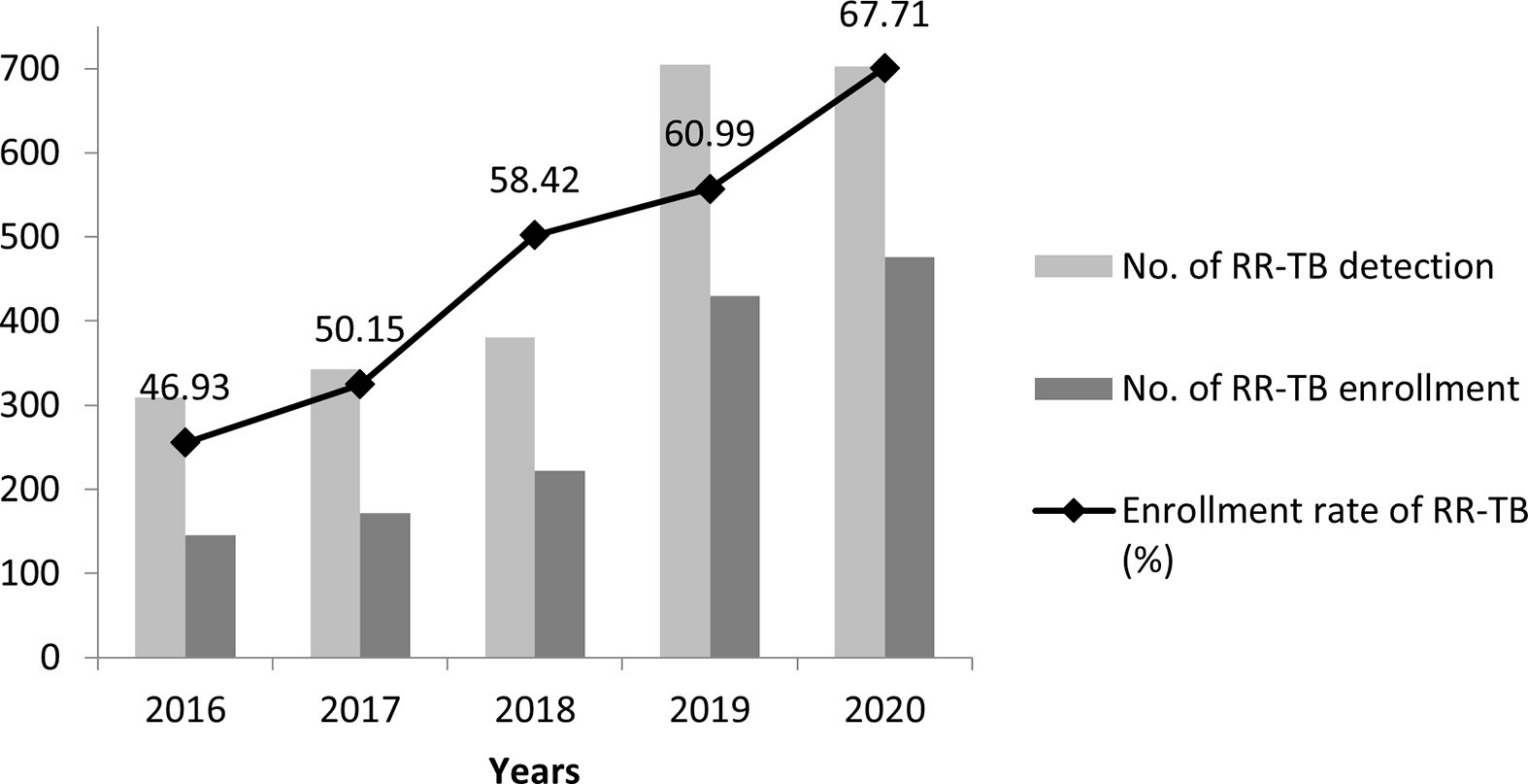
Distribution of enrollment changes for RR-TB in Yunnan, 2016–2020. Number of RR-TB patients detected refers to those detected using molecular tests or conventional DST.—Number of RR-TB patients enrolled refers to those enrolled by RR-TB care facilities.—Enrollment rate of RR-TB (%) refers to the number of enrolled RR-TB patients divided by the number of all the detected RR-TB patients. * The trend chi square test showed that enrollment rate of RR-TB in 2016–2020 was statistically different.

Yunnan saw a trend of steady increases in enrollment in treatment of RR-TB between 2016 and 2020. No more than 60.0% of the detected RR-TB patients were enrolled in treatment from 2016 to 2018. In contrast, treatment enrollment in 2020 was increased up to 67.7% (χ^2^_trend_ = 51.9, *P*<0.001), which may have resulted from the major measures taken between 2018 and 2020, including the scaled up provincial efforts to facilitate increased enrollment of RR-TB patients by designated RR-TB care facilities, strengthening capacity of the cadre of personnel of TB prevention and control, provision of patient-centered care and introduction of short course regimen. Data from this study, however, suggested a significant treatment gap with 32.3% of the RR-TB patients identified were not initiated on the standardized RR-TB treatment in 2020 (Fig 2).

The data showed that, out of the 995 RR-TB patients not enrolled in treatment, 108 (10.9%) died after diagnosis and before starting RR-TB treatment, 21 (2.1%) were lost to follow up; 639 (64.2%) refused treatment due to financial difficulties; 89 (8.9%) continued using first-line anti-TB treatment because of patients’ refusal to change or care providers’ false belief based on their clinical experience that conditions of some RR-TB patients could be improved by using the first-line therapy; 9 (0.9%) could not be treated with appropriate RR-TB regimen based on assessment; and 129 (13.0%) were transferred out to other places. From 2016 to 2020, the main reason for non-enrollment is patient refusal of treatment due to financial difficulties (which accounted for more than 50.0% of the RR-TB patients not enrolled in treatment consistently across the years, although it varied between 54.0% and 71.0%) followed by death and loss to follow-up.

Among the 2440 patients diagnosed between 2016 and 2020, 62.2% of the females (686) and 58.0% of the males (1754) were enrolled on treatment. However, there was no significant difference in treatment enrollment between males and females (*P* = 0.057). Comparison between the age groups reveal that the elderly patients aged 65 years old or above tended to be more likely to interrupt treatment than the younger ones (*P*<0.001). Although treatment enrollment among the Han Chinese patients (60.2%) was slightly higher than that in the patients from ethnic minorities (57.3%), the difference was not statistically significant (*P* = 0.166). Among the 2,034 patients who were farmers, rural migrant or factory workers, 56.7% were initiated on treatment. In contrast, more than 70% of the patients in the groups of children/students/teachers, management cadre/medical staff started RR-TB treatment. There were 881 re-treated patients, who were defined as those who had been exposed to anti-TB treatment for more than one month prior to the current diagnosis of RR-TB. Among all the re-treated patients, 64.1% were initiated on treatment of RR-TB, which indicates a significantly higher level of treatment enrollment than in the new patients (*P*<0.001). In comparison of use of the two options available for detection of rifampin resistance, there was a significant difference in the rate of patient enrollment between the two groups of patients who used conventional Drug Susceptibility Testing (DST) (that generally takes 2–3 months to report the result) or GeneXpert MTB/RIF (that can give results in 2–4 hours only, significantly reducing the delay to diagnosis of RR-TB) (*P*<0.001). Treatment enrollment may vary according to which level of TB care facilities providing diagnosis of RR-TB. Data suggests that treatment enrolment of RR-TB patients was significantly higher in provincial medical institutions than in the lower, prefectural-level TB care facilities (*P*<0.001) (Table 2).

**Table 2:** Characteristics among enrollment and non-enrollment RR-TB treatment groups (n = 2440).

Characteristics	Total (n = 2440)		Number of RR-TB patients enrolled (n = 1445) n (%)		Number of RR-TB patients not enrolled (n = 995) n (%)	
**Gender**						
Male		1754		1018 (58.0)		736 (42.0)
Female		686		427 (62.2)		259 (37.8)
**Age group (years)** *						
15–34		608		413 (67.9)		195 (32.1)
35–64		1484		888 (59.8)		596 (40.2)
≥65		348		144 (41.4)		204 (58.6)
**Ethnicity**						
Han		1594		960 (60.2)		634 (39.8)
Minority		846		485 (57.3)		361 (42.7)
**Occupation**						
Children/Students/Teachers		98		75 (76.5)		23 (23.5)
Management cadre/medical staff		42		38 (90.5)		4 (9.5)
Commercial service personnel		24		15 (62.5)		9 (37.5)
Retired personnel		57		31 (54.4)		26 (45.6)
Farmer/rural migrants/factory worker		2034		1154 (56.7)		880 (43.3)
Others		185		132 (71.4)		53 (28.6)
**Patient type** *						
New		1065		564 (53.0)		501 (47.0)
Re-treatment		1375		881 (64.1)		494 (35.9)
**DST** *						
Molecular test		1603		1032 (64.4)		571 (35.6)
Conventional DST		837		413 (49.3)		424 (50.7)
**Level of TB care facility** *						
Provincial		1058		831 (78.5)		227 (21.5)
Prefecture/municipal		1382		614 (44.4)		768 (55.6)

New patients refer to the patients who have never been exposed to anti-TB treatment or have not received anti-TB treatment for more than one month.

Re-treatment refers to the TB patients who had been exposed to anti-TB treatment in the past or relapse after cure or were retreated without reaching cure.

* Single-factor chi-square test showed that these variables were statistically different.

Binary logistic regression analysis was applied to examine the factors that may have influenced patient enrolment in treatment of RR-TB in Yunnan between 2016 and 2020. It showed statistically significant results in the three key risk factors—age, diagnosis by DST and patient enrollment by level of TB care facility. The patients who were aged 65 or above, tested using conventional DST, diagnosed by the prefecture and municipal-level RR-TB care facilities were less likely to enroll in RR-TB treatment. The protective factor was patient type, which means that those with previous TB treatment were more likely to enroll in RR-TB treatment. In conclusion, non-enrollment for RR-TB was higher among the patients who were aged 65 or above, re-treated, tested using conventional DST or diagnosed by the prefecture- and municipal-level RR-TB care facilities (Table 3).

**Table 3:** Independent risk factors for treatment enrollment of RR-TB identified by multivariate model.

Factors	Total (n = 2440)	Number of RR-TB non-enrollment (n = 995)	aOR	95%CI	*P* value
**Age group (years)**					
15–34	608	195	1.0	Ref*	
35–64	1484	596	1.2	(0.948–1.469)	0.138
≥65	348	204	2.7	(1.997–3.614)	<0.001
**Patient type**					
New case	1065	501	1.0	Ref	
Re-treatment	1375	494	0.7	(0.607–0.867)	<0.001
**DST**					
Molecular test	1603	571	1.0	Ref	
Conventional DST	837	424	1.9	(1.620–2.344)	<0.001
**Level of TB care facility**					
Provincial	1058	227	1.0	Ref	
Prefecture and municipal	1382	768	4.4	(3.608–5.250)	<0.001

aOR = Adjusted odds ratio.

95% = CI 95% confidence interval.

*P*-value: The difference is statistically significant when the *P*-value is less than 0.05.

* Reference.

## Discussion

The data in the TBMIS showed a low level of screening and enrollment of RR-TB patients in Yunnan in 2016, which was similar to the finding from another study in Yichang, China [6]. From 2017 to 2020, a patient-centered, comprehensive model of RR-TB prevention and control had been established in Yunnan and achieved significant outcomes, including streamlined algorithms for rapid detection and clinical care of RR-TB, strengthened capacities and increased resources for TB laboratories, scaled up use of rapid molecular diagnostics, and patient-centered care and counselling along key points of cascade of RR-TB care. Strengthened RR-TB response resulted in improved screening (93.8%) and detection (70.3%) of RR-TB in Yunnan. In addition, the turnaround time for diagnosis of RR-TB has been effectively reduced. A study in Quzhou showed use of Xpert MTB/RIF reduced time to diagnosis (3.2 days) [11]. As of the writing of this paper, the median diagnostic delay in Yunnan had been reduced to one single day, which means that, in each county (district) of Yunnan, RR-TB patients can be detected within 24 hours, and tracked and followed up immediately by the local CDCs to enable timely enrollment in treatment at the higher-level RR-TB care facilities so as to minimize the spread of undetected RR-TB in hard-to-reach patients in the community.

From 2016 to 2020, approximately 50% on average of the RR-TB patients in Yunnan were not enrolled in treatment, and 64.2% (639 cases) refused treatment among them because they could not afford to pay for it. A similar situation was seen in a study in Hunan in 2016 which revealed the most frequently reported reason for non-enrollment among 482 RR-TB patients was financial hardship (111 cases, 23.0%) [12]. Ying Liu identified the main reason for non-enrollment in Chongqing that, 41.94% (26 cases) of the confirmed RR-TB patients from 2009 to 2012 reported they could not afford to pay for the high-cost second-line anti-TB drugs [13]. This result resonates with the findings on the implication of financial burden on the treatment enrollment of RR-TB from a study by Li Zhipeng in Xuzhou and Ganzhou from 2011 to 2017 [14]. In China, the family income of 82.0% of the TB patients is lower than the national average, and the economic burden of RR-TB patients is even higher than that of drug-susceptible TB patients [15]. In Yunnan, drug-susceptible TB patients are provided with anti-TB drugs free through the National Tuberculosis Control Fund while RR-TB patients have to pay for their anti-TB treatment through medical insurance scheme and/or out of their pockets. According to a study in Yunnan by Hutchison in 2017, RR-TB patients and health care professionals reported financial constraints as “the most pervasive barrier to care”, and barriers could be greater among ethnic minorities and patients coming from rural areas, especially those with RR-TB [16]. The implication of the RR-TB patients’ medical insurance on treatment enrollment was unknown because medical insurance was not considered for data collection in this research and should be further examined in future studies.

Our data showed that, at the time diagnosis of RR-TB was confirmed, 10.9% of the RR-TB patients died and 2.1% were lost to follow-up. A study in South Africa reported that 11.6% of the RR-TB patients died before treatment initiation [17]. Multivariate analysis in this study highlighted that the laboratory diagnostic methodology use affected treatment enrollment for RR-TB, as non-enrollment was higher among the RR-TB cases detected by conventional DST versus rapid molecular testing. The turnaround time for RR-TB diagnosis in Yunnan in 2016 was at least 2–3 months on average because conventional DST based on solid medium was adopted. The condition of RR-TB patients could deteriorate significantly during the long diagnostic delay. Therefore, use of molecular diagnostic tests for rapid detection of drug resistance, such as Xpert MTB/RIF, should be scaled up [18]. As recommended in the 2020 national guidelines on standard practices for TB control and prevention by China Center of Disease Control and Prevention, molecular testing for screening of RR-TB is the preferred option for the TB care facilities equipped with molecular diagnostics to detect drug resistance [19]. As of the writing of this paper, molecular diagnostic testing was provided free to the bacteriologically positive patients for RR-TB screening through the National TB Control Fund in Yunnan. In order to scale up the use of the high-cost molecular diagnostic tests for rapid detection of drug resistance, we suggested that it should be covered by the medical insurance scheme in the long term.

In this study, the probability of enrollment was lower among elderly patients over 65 years old. This conclusion was echoed by Feng Lu [7]. Leilei Zhang’s research shows that the situation of elderly RR-TB patients can be confounded with other comorbidities, which may exacerbate intolerance to some RR-TB treatment regimens [20]. In 2018, an unpublished qualitative study in Yunnan as part of the CAP-TB project examined the barriers to enrolment in treatment of multidrug-resistant TB and found that clinical complications and lack of self-care ability among the elderly meant that many RR-TB elderly patients did not initiate RR-TB treatment.

Similar to the findings in Hunan, China, the study suggested that, compared with new patients, retreatment patients are more likely to enroll because of their better awareness based on previous personal experiences [12].

While the above findings were in line with previous studies, the following were found to be unique in the resource limited social settings like Yunnan. This study suggested statistical difference in enrollment of RR-TB patients diagnosed by different levels of TB care facilities. The prefecture and municipal-level TB care facilities, although more conveniently accessible for patients, enrolled a limited number of patients while the provincial-level facilities received a higher volume of patients for care. On the one hand, the prefecture- and municipal-level TB care facilities, in spite of the evaluation and accreditation in 2016 and 2017, were not fully prepared to enroll all local RR-TB patients as required by the provincial government. Treatment enrollment at the prefecture and municipal level was constrained by a lack of laboratory equipment, limited physician capacity to diagnose and manage RR-TB properly, and insufficient drug supply. On the other hand, there was a shared norm among the patients that provincial-level hospitals are comprehensively and sophisticatedly equipped and have stronger clinical capacity, in comparison with the lower-level TB care facilities. Many patients, therefore, made hard choice of paying more than they could afford and travelling long journeys to the capital city for regularly scheduled clinical visits and drug refill. This issue needs to be properly addressed in order to effectively improve treatment enrollment of RR-TB in the long term.

## Conclusion

RR-TB is more challenging to treat due to resistance to core first-line anti-TB drugs and requirements for complex diagnostics, longer treatment course and more costly second-line drugs. Implementation of *the 13th Five-Year Plan for TB Prevention and Control* resulted in success of a comprehensive model for RR-TB care featured by patient-centered care. Yunnan saw a paradigm shift in its TB response strategy with more attention placed on the needs and perspectives of the demand (RR-TB patients), which resulted improved screening, detection and treatment enrollment of RR-TB. This study confirmed that scaled up use of molecular diagnostics significantly increased screening of RR-TB and led to enrollment of more RR-TB patients in the end. It also suggests that targeted interventions are needed to tackle the major barriers that lie along the cascade of RR-TB care, including streamlined algorithm for diagnosis of RR-TB to reduce diagnostic delay, strengthened clinical training for prefecture- and municipal-level care providers, effective patient education and counselling at the point of diagnosis to strengthen well-informed decisions made by the patients, and follow-up support to help patients overcome difficulties and adhere to RR-TB treatment, especially for those elderly patients who are lack of self-care ability and can hardly travel alone. To close the gaps and improve treatment enrollment in the long run, we should strengthen the delivery of comprehensive, patient-centered care with greater multi-sectoral engagement, including finance, social welfare and labor.
